# Mechanistic Insights into Regulation of JAK2 Tyrosine Kinase

**DOI:** 10.3389/fendo.2017.00361

**Published:** 2018-01-05

**Authors:** Stevan R. Hubbard

**Affiliations:** ^1^Department of Biochemistry and Molecular Pharmacology, Skirball Institute of Biomolecular Medicine, New York University School of Medicine, New York, NY, United States

**Keywords:** Janus kinases, protein tyrosine kinases, cytokine receptor, autoinhibition, cell signaling and regulation

## Abstract

JAK2 is a member of the Janus kinase (JAKs) family of non-receptor protein tyrosine kinases, which includes JAK1–3 and TYK2. JAKs serve as the cytoplasmic signaling components of cytokine receptors and are activated through cytokine-mediated *trans*-phosphorylation, which leads to receptor phosphorylation and recruitment and phosphorylation of signal transducer and activator of transcription (STAT) proteins. JAKs are unique among tyrosine kinases in that they possess a pseudokinase domain, which is just upstream of the C-terminal tyrosine kinase domain. A wealth of biochemical and clinical data have established that the pseudokinase domain of JAKs is crucial for maintaining a low basal (absence of cytokine) level of tyrosine kinase activity. In particular, gain-of-function mutations in the *JAK* genes, most frequently, V617F in the pseudokinase domain of JAK2, have been mapped in patients with blood disorders, including myeloproliferative neoplasms and leukemias. Recent structural and biochemical studies have begun to decipher the molecular mechanisms that maintain the basal, low-activity state of JAKs and that, *via* mutation, lead to constitutive activity and disease. This review will examine these mechanisms and describe how this knowledge could potentially inform drug development efforts aimed at obtaining a mutant (V617F)-selective inhibitor of JAK2.

## Introduction

Janus kinases (JAKs) are non-receptor protein tyrosine kinases that serve as the catalytic signaling components for a wide range of cytokine receptors, including the receptors for interleukins, interferons, growth hormone, erythropoietin, and leptin (1). There are four mammalian members of the JAK family: JAK1-3 and TYK2 (tyrosine kinase-2), which are constitutively bound to the cytoplasmic region of cytokine receptors. In general, binding of cytokines to the extracellular region of their cognate receptors induces receptor dimerization, facilitating *trans*-phosphorylation (activation) of the associated JAKs. Typically, the dimerized receptor chains are distinct (e.g., interferon-α receptors 1 and 2) and harbor two different JAK molecules (e.g., JAK1 and TYK2) (2). Cytokine receptors belonging to a subclass that includes erythropoietin receptor (EpoR) and growth hormone receptor (GHR) are homodimeric and bind JAK2 exclusively. For these receptors, it is not entirely clear whether they are dimerized by cytokine or exist as pre-formed, inactive dimers that undergo a cytokine-induced structural rearrangement. Activated JAKs phosphorylate specific tyrosine residues on the cytokine receptors and subsequently on signal transducer and activator of transcription (STAT) proteins (3), which are recruited to the phosphorylated receptors through their SH2 (Src-homology 2) domains. Phosphorylated STATs then translocate to the nucleus to initiate specific transcriptional programs. JAK–STAT signaling pathways are critical for organismal development and homeostasis, particularly in immunity (1, 3).

**Figure 1:**
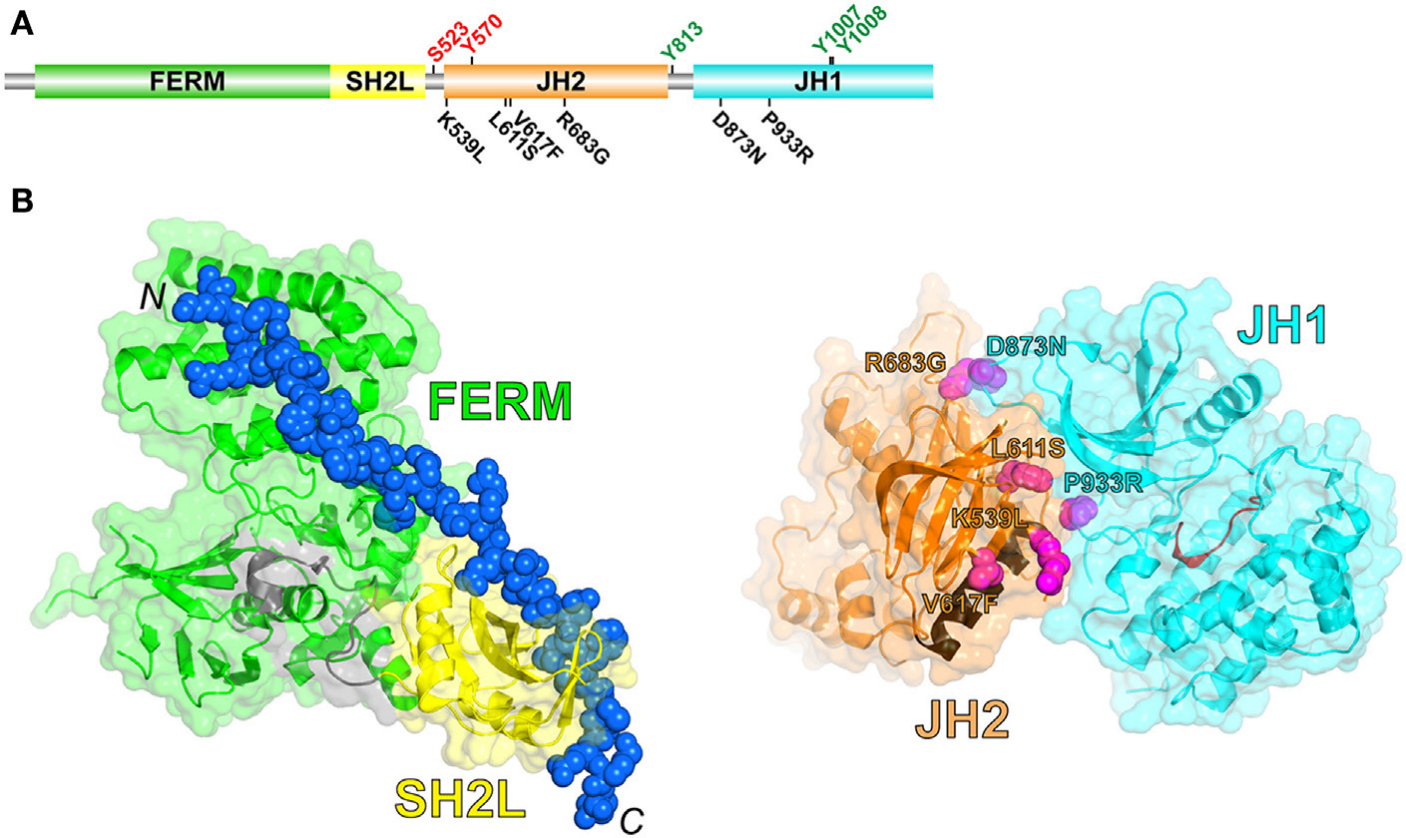
**(A)** Schematic diagram of the domain organization in JAK2, shown to linear scale (1132 residues in human JAK2). Positive- and negative-regulatory phosphorylation sites (colored green and red, respectively) whose mechanisms are understood are shown above the domains. Select activating mutations in JH2 and JH1 are shown below the domains. **(B)** (Left) Crystal structure of the JAK1 FERM and SH2L domains in complex with a peptide representing the interferon-λ1 receptor (6) (PDB code 5L04). The FERM domain is colored green, the SH2L domain is colored yellow, the SH2L-JH2 linker is colored gray, and the receptor peptide (shown in sphere representation; the N- and C-termini are labeled) is colored blue. (Right) Crystal structure of JH2 and JH1 of TYK2 (8) (PDB code 4OLI). JH2 is colored orange, with the C helix colored brown, and JH1 is colored cyan, with the catalytic loop colored red. The positions of select activating mutations in JAK2, corresponding to those in **(A)**, are shown in sphere representation and colored magenta (TYK2 residues shown, labeled according to the JAK2 mutations).

JAKs possess four structural domains (Figure 1A): an N-terminal FERM (band 4.1, ezrin, radixin, moesin) domain, an SH2-like (SH2L) domain, a kinase-like or pseudokinase domain [JH2 (Janus homology-2)], and a C-terminal tyrosine kinase domain (JH1). The FERM and SH2L domains form a single structural unit that engages the so-called Box1 and Box2 cytoplasmic regions of cytokine receptors (4) (Figure 1B, *left*). The SH2L domain of JAKs binds, in its aberrant phosphotyrosine-binding pocket, a glutamate residue in the Box2 region of the receptor (4–6). As far as is known, receptor binding and specificity are determined solely by the FERM and SH2L domains of JAKs, although the specificity determinants are not fully understood (7).

While homozygous loss-of-function mutations in *JAK* genes can be lethal (*JAK1* and *JAK2*), or highly debilitating (*JAK3*, severe combined immunodeficiency), heterozygous gain-of-function mutations in *JAK* genes can give rise to blood disorders known as myeloproliferative neoplasms (MPNs), which include polycythemia vera, primary myelofibrosis, and essential thrombocythemia, and also to leukemias (9). In a series of papers published in 2005 (10–13), a single point mutation in the pseudokinase domain of JAK2, V617F, was identified in >90% of patients with polycythemia vera and in ~50% of patients with primary myelofibrosis and essential thrombocythemia. Other point mutations, mainly in the pseudokinase domains of JAKs, have also been linked to proliferative blood disorders (14, 15).

## Regulation of JAK2 Activity

The tyrosine kinase domain (JH1) of JAKs is responsible for the majority, if not all, of the phosphoryl transfer activity of JAKs (see below regarding JAK2 JH2 activity). In particular, JH1 catalyzes *trans*-phosphorylation of two tyrosine residues in the kinase activation loop (Tyr1007 and Tyr1008 in JAK2), which stabilizes the active state. *Trans*-phosphorylation of these two tyrosines is the key step in JAK activation. The activated kinase domain then phosphorylates specific tyrosine residues in the associated cytokine receptor and in the recruited STAT molecules, as well as other tyrosines in the JAK molecule (Figure 1A).

### The JAK2 Pseudokinase Domain Negatively Regulates Tyrosine Kinase Activity in the Basal State

Biochemical studies, in addition to sequencing data from MPN patients mentioned above, have implicated the pseudokinase domain (JH2) of JAKs as a negative regulator of the tyrosine kinase activity of JH1 (16–18) [reviewed in Ref. (19)]. In 2014, the long-sought autoinhibitory interaction between JH2 and JH1 was elucidated by X-ray crystallography [TYK2 (8)] and, independently, by molecular dynamic simulations [JAK2 (20)]. In this intramolecular (*cis*) interaction, the N lobe of JH2 interacts with the N lobe and hinge region of JH1 (Figure 1B, *right*). Importantly, nearly all of the mapped activating mutations in JH2 (e.g., R683G, L611S) and JH1 (e.g., D873N, P933R) lie in this interface, consistent with the hypothesis that destabilization of this interface leads to an increase in JAK2 activation through increased *trans*-phosphorylation of JH1. (Conspicuously, V617F is not found in the interface and is discussed below.) How this interaction with JH2 suppresses the kinase activity of JH1 is not completely clear, but probably involves physical sequestration of JH1 and/or constraints on JH1 lobe movements that are necessary for phosphoryl transfer activity.

In addition to functioning sterically as a negative regulator of JH1, JAK2 JH2 was shown to possess weak catalytic activity (21), phosphorylating two sites, Ser523 and Tyr570, which had previously been identified as negative-regulatory phosphorylation sites (22–25). It is not well understood how Ser523 and Tyr570 are phosphorylated by JAK2 JH2. According to the crystal structure (26), the end of the activation loop, which forms a short α helix, would seem to impede substrate access to the active site. That is, some rearrangement of the activation loop would be necessary for access. Ser523 was shown to be constitutively phosphorylated in cells (24), whereas Tyr570 has low, but significant, basal phosphorylation, which increases upon cytokine stimulation (22, 23), indicating that JH1 probably also phosphorylates Tyr570 as a negative-feedback mechanism. *In vitro* studies of JAK2 JH2-JH1 also implicate JH1 as the key kinase domain for phosphorylation of Tyr570 (27). Taken together, these studies suggest that, *in vivo*, JH2 is responsible for Ser523 phosphorylation and that JH1 is primarily responsible for Tyr570 phosphorylation.

There are no reports of JH2 catalytic activity for the other three JAKs, possibly because there are no sites to phosphorylate; Ser523 and Tyr570 are not conserved in JAKs. However, biochemical and structural studies have shown that JAK1, JAK2, and TYK2 are all capable of binding Mg-ATP (28, 29). Interestingly, JAK3 contains a lysine (Lys652) in place of the conserved asparagine residue at the end of the catalytic loop. This asparagine in JAK2 and TYK2 JH2, and in *bona fide* protein kinases, coordinates the ATP-associated Mg^2+^ ion (26, 29), and a lysine at this position might bind to the phosphates of ATP directly, bypassing the requirement for Mg^2+^. Thus, JAK3 probably binds ATP but without a divalent cation.

Regarding the negative regulatory roles of pSer523 and pTyr570, the model for JAK2 JH2-JH1 derived from molecular dynamics simulations (20) suggests that pSer523 and pTyr570 interact with positively charged residues in JH1 to stabilize the JH2–JH1 autoinhibitory interaction. In general, for JAKs, the β2–β3 loop of JH2, where pTyr570 in JAK2 resides, is negatively charged, and the N lobe of JH1 (interaction site for pTyr570) is positively charged, suggesting that a favorable charge interaction between these two regions stabilizes the JH2–JH1 autoinhibitory interaction for all JAKs.

### The JAK2 Pseudokinase Domain Positively Regulates Constitutive Tyrosine Kinase Activity in Pathologic Signaling

Several biochemical studies have revealed that the role of JAK2 JH2 in pathologic activation (by point mutation) is complex. That is, loss of the autoinhibitory interaction between JH2 and JH1 through mutation (e.g., R683G in JH2) is necessary but not sufficient for constitutive activation of JAK2. The first evidence for this came from mutation of Phe595 (to alanine) in the C helix of JH2, which suppressed the hyperactivity of V617F (30). Suppression of V617F activity by F595A could be explained by the close spatial relationship (π stacking) between these two phenyalanines (26). Thus, replacement of Phe595 by a less bulky alanine could potentially restore the wild-type local conformation and hence the JH2-JH1 autoinhibitory interaction. However, in the same study (30), F595A also suppressed other activating mutations, for example, R683G, which is ~27 Å from Phe595. This suggests that F595A suppression of R683G (and V617F) hyperactivation is probably due to destabilization of the C helix. In addition, mutations in the ATP binding pocket of JH2 (which abrogate ATP binding) also suppressed the hyperactivity of various activating mutations (31), presumably because JH2 is less stable without nucleotide bound. Finally, F739R, which is predicted to disrupt the C lobe of JH2 (if not JH2 entirely), also suppressed V617F hyperactivity (26). These mutagenesis data are consistent with a positive role for JH2 in the pathologic activation of JAK2 by V617F and other activating mutations, and demonstrate that an activating point mutation in JH2 destabilizes the JH2–JH1 autoinhibitory interaction without compromising the structural integrity of JH2, particularly in the C-helix region.

**Figure 2:**
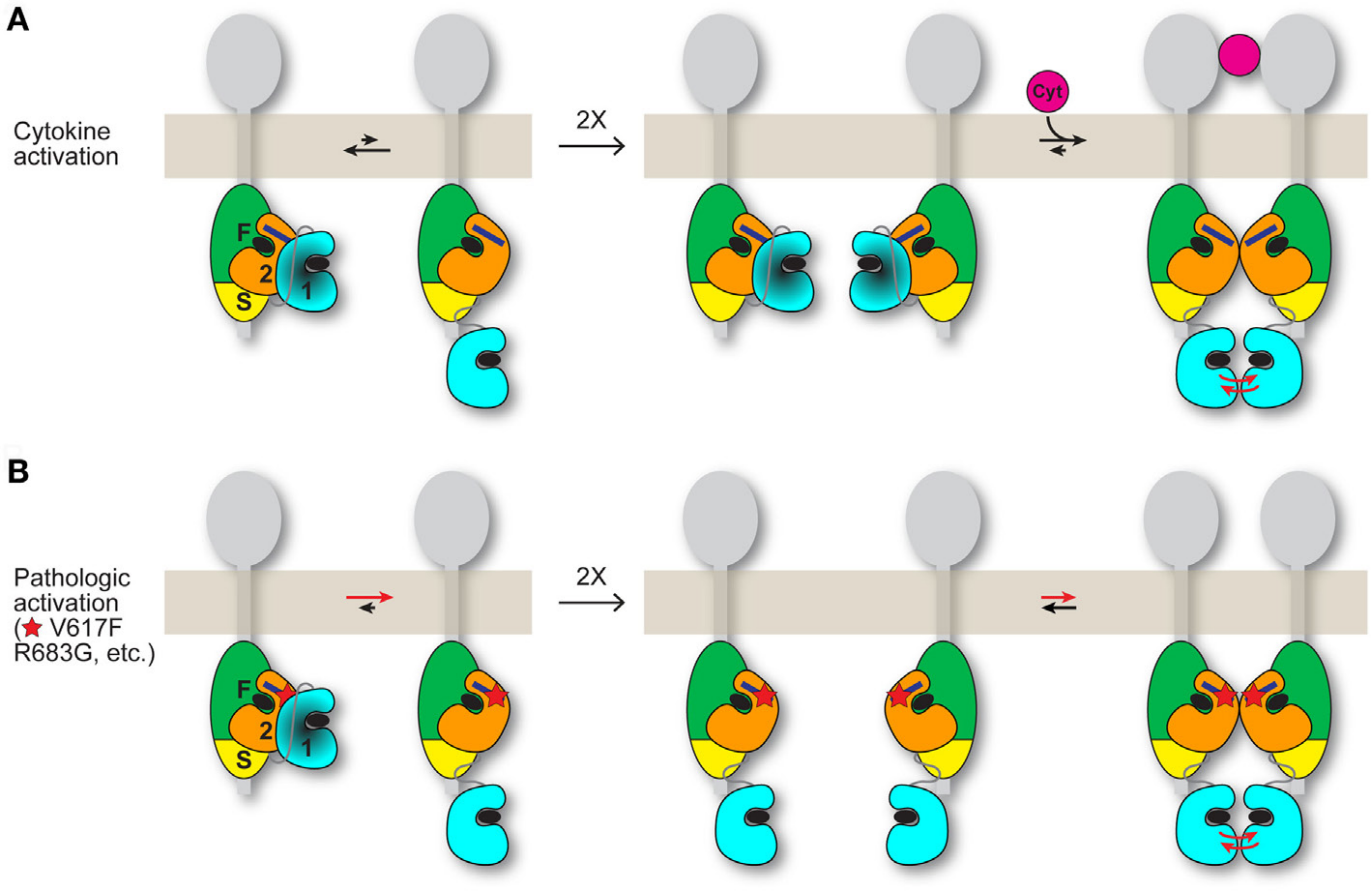
Possible models for JAK2 activation by cytokine and by activating mutation. JAK2 is associated with the cytoplasmic region of a cytokine receptor (gray). The four domains of JAK2 are labeled “F” for FERM, “S” for SH2L, “2” for JH2, and “1” for JH1. The integrated FERM and SH2L domains bind to the cytokine receptor. How JH2–JH1 interacts with FERM-SH2L is not known. The C helix of JH2 is represented by a dark blue rectangle, and ATP is represented by a black ellipse. **(A)** (Left) An equilibrium (black arrows) exists between the autoinhibited state, in which JH2 exerts an autoinhibitory interaction on JH1 (darkened), and a state in which JH1 is disengaged from JH2 and is phosphorylation-competent, with the autoinhibitory state favored for wild-type JAK2. (Middle) In the absence of cytokine, *trans*-phosphorylation of autoinhibited JH1 is limited. (Right) Binding of a cytokine (magenta) to the extracellular region of the receptor induces receptor dimerization, which promotes *trans*-phosphorylation of JH1 (red arrows). The dimerization process might also include formation of a JAK2 dimer, shown here as a JH2 dimer. **(B)** (Left) Activating point mutations (shown as a red star) such as V617F and R683G in JH2 shift the equilibrium from the autoinhibitory state to the phosphorylation-competent (and JAK2 dimerization-competent) state. (Middle and right) Mutant JAK2 is capable of dimerizing in the absence of cytokine. Overlap is predicted between the interfaces used by JH2 for JH1 autoinhibition and for JH2-mediated JAK2 dimerization. The activating mutation may directly disrupt the autoinhibitory interaction (e.g., R683G) or hyperstabilize the JAK2 dimer, or both (possibly V617F). Mutations that destabilize the domain structure of JH2, such as those in the ATP binding pocket (31), would suppress activating mutations (V617F, etc.) by destabilizing JAK2 dimer formation, which is necessary for activation in the absence of cytokine. Whether the putative JH2-mediated JAK2 dimer required for mutational activation in the basal state **(B)** is the same JAK2 dimer that may form when cytokine dimerizes the receptors **(A)** is not known. If homodimeric receptors such as EpoR are pre-dimerized in the basal state, then cytokine binding would rearrange the dimer (instead of dimerizing monomeric receptor-JAK2 pairs) to juxtapose the two kinase domains for *trans*-phosphorylation **(A)**, possibly facilitated by a JH2-mediated interaction. An activating mutation would promote the receptor-JAK2 rearrangement through JH2-mediated JAK2 dimerization in the absence of cytokine binding **(B)**.

A plausible mechanism for the positive role played by JH2 in pathologic activation is that, when JH2 autoinhibition of JH1 (in *cis*) is weakened by mutation, JH2 mediates dimerization of JAK2 in the absence of cytokine, which facilitates JH1 *trans*-phosphorylation (Figure 2). In essence, pathologic activation of JAK2 might rely on JH2-mediated JAK2 dimerization (cytoplasmic) rather than cytokine-mediated receptor dimerization (extracellular). Although JAK2 JH2 and JH2–JH1 proteins harboring activating mutations such as V617F are monomeric in solution (26, 27, 32), full-length JAK2 associated with transmembrane cytokine receptors might behave differently. Receptor-associated JAK2 undergoes quasi-two-dimensional diffusion on the cell surface with some degree of molecular orientation, versus three-dimensional diffusion in the cytoplasm (with random orientation) without receptor. The JAK2 dimerization propensity would be substantially higher in the former case. Indeed, the requirement for cytokine receptor (and for FERM-SH2L, which engages the receptor) for JAK2 V617F hyperactivity (33) can be readily explained by this difference in diffusion properties.

With regard to the nature of the putative JH2-mediated JAK2 dimer, because JH1 of JAK2 (and of other JAKs) has relatively high activity on its own (8, 34, 35), it is unlikely that JH2 exerts an allosteric effect on JH1 in *trans* (or in *cis*), although an allosteric activation mechanism in *cis* between JH2 V617F and JH1 has been proposed (36). Moreover, JH2 would probably not interact with JH1 in *trans* as it does in *cis* (autoinhibitory interaction) due to the presence of the activating mutation (which weakens the JH2-JH1 autoinhibitory interaction) and possibly to steric constraints. Therefore, the positive interaction mediated by JH2 probably involves either JH2 or FERM-SH2L in the other JAK2 molecule (Figure 2). Structural and biochemical evidence point to the C helix as the likely site of interaction on JH2 (26, 30, 36). Although a JH2 dimer was observed in the crystal structure of JAK1 JH2 (32), in which, interestingly, the C helix was in the center of the dimer interface, mutagenesis studies performed on JAK2 in that study did not support a role for this particular JH2 dimer in activation by V617F.

### Possible Mechanisms for Pathologic JAK2 Activation by V617F

As mentioned above, Val617 in JH2, the site of the activating mutation (V617F) responsible for the majority of MPNs, is not situated in the JH2–JH1 autoinhibitory interface. The molecular dynamics-based model of the JH2–JH1 autoinhibitory interaction (20) suggests that Val617 is near the SH2L-JH2 linker, which in the model is wedged between JH2 and JH1. The SH2L-JH2 linker was shown to play a role in maintaining JAK2 basal activity (37). Therefore, it is conceivable that substitution of Val617 with bulky residues could displace this linker, decreasing the stability of the JH2–JH1 autoinhibitory interaction. While this may contribute to the constitutive activity of V617F, it is probably not the main activating mechanism. Moreover, *in vitro* biochemical studies of the tandem kinase domains (JH2–JH1) of JAK2 (27) or TYK2 (8) demonstrated that V617F (V658F in TYK2) does not increase the intrinsic catalytic activity of JH1.

Based on the crystal structures of wild-type JAK1 JH2 and V658F (V617F-equivalent), Eck and colleagues (32) hypothesized that V617F induces a switch in the conformation of Phe537, which is at the beginning of JH2, from a tucked position near Phe595 in the C helix to an exposed position. How this conformational switch would lead to activation of JAK2 is not understood, though.

An attractive hypothesis for the mechanism of JAK2 activation *via* V617F is that the mutation hyperstabilizes the putative JH2-mediated JAK2 dimer discussed above (Figure 2), resulting in efficient JH1 *trans*-phosphorylation. Some support for this mechanism comes from cell-transfection experiments of JAK2 (with two different tags), wild-type or V617F, in which only V617F showed evidence of co-immunoprecipitation (38). Other activating mutations such as R683G, which directly destabilize the JH2–JH1 autoinhibitory interaction, also depend on a structurally sound JH2 (discussed above), but whether V617F and R683G, etc. potentiate formation of the same JAK2 dimer or distinct ones is not clear. That E596R in the C helix of JAK2 JH2 suppresses the hyperactivation of V617F but not of R683G or K539L (36) hints that the dimers might be distinct.

## Outstanding Mechanistic Issues

Many important mechanistic questions regarding JAK2 activation, both by normal cytokine stimulation and by pathologic mutation, remain unanswered. For example, are class I homodimeric cytokine receptors such as GHR, EpoR, and thrombopoeitin receptor pre-dimerized on the cell surface in the basal state (with non-activated JAK2)? For pre-dimerized receptors, cytokine binding would re-arrange two relatively closely apposed receptor-JAK2 pairs, stimulating JAK2 *trans*-phosphorylation. Most studies have focused on EpoR, and biochemical evidence for pre-dimerized receptors has been reported (39, 40), yet, in the one study that employed in-cell single-molecule fluorescence techniques, EpoR dimers in the basal state were not observed (41). The cell-surface density of expressed receptors is obviously a key parameter in oligomerization studies.

If homodimeric receptors are pre-dimerized in the basal state, an additional question arises whether autoinhibition of JH1 by JH2 occurs in *cis* (same JAK2 molecule) or in *trans* (between JAK2 molecules), evidence for the latter (and for pre-dimerized GHR) was reported for JAK2 on GHR (42). Because the JH2-JH1 linker is relatively long (~30 residues in JAK2 and JAK3, ~15 residues in JAK1 and TYK2), the JH2–JH1 interaction detailed above (Figure 1B, *right*) could potentially occur in *trans* rather than in *cis*. [In the TYK2 JH2–JH1 crystal structure (8), there is a slight c*is*/*trans* ambiguity because of the disordered JH2–JH1 linker.] However, the equivalent JAK2 V617F mutations in JAK1 (V658F) and TYK2 (V678F) are also activating, and because JAK1 and TYK2 reside on monomeric receptors in the basal state, the JH2–JH1 interaction would necessarily be in *cis* for these two JAKs.

Another outstanding question is the nature of the four-domain structure of JAKs in the basal (autoinhibited) state. We know how the FERM and SH2L domains are structurally integrated to bind to cytokine receptors (7) (Figure 1B, *left*) and how JH2 interacts with JH1 (8, 20) (Figure 1B, *right*), but we do not know the spatial relationship between FERM-SH2L and JH2–JH1. JAK2 JH2–JH1 as expressed in insect cells is active (27), suggesting that autoinhibition requires all four domains. Low-resolution electron micrographs of full-length JAK1 (negative-stained) indicate that the four-domain structure may be loosely assembled (43), which is probably important for the transitioning of JAKs from an autoinhibited state to a phosphorylation-competent state. Finally, the exact mechanisms by which V617F and other activating mutations lead to constitutive JAK2 activity remain to be determined.

## Prospects for a Mutant JAK2 Inhibitor

Knowledge of the mechanisms by which JAK2 is activated through normal cytokine binding and by mutation could inform our efforts to design a mutant (V617F)-specific JAK2 inhibitor for the treatment of MPNs. Currently, there is one drug in the clinic, ruxolitinib (Jakafi^®^), for the treatment of primary myelofibrosis and polycythemia vera. Ruxolitinib is a JAK2 (and JAK1) small-molecule inhibitor that binds in the ATP binding pocket of JH1. As such, it inhibits the catalytic activity of wild-type JAK2 as well as mutant JAK2, which can lead to side effects such as anemia and thrombocytopenia.

How to target for inhibition JAK2 molecules harboring mutations in JH2 (e.g., V617F) rather than in JH1 poses a difficult conceptual and practical problem (44). If V617F and proximal residues in the C helix of JH2 are involved in an activating *trans* interaction (as discussed above), it may be possible to disrupt this interaction with a small molecule that binds to this region, although this region is rather shallow and thus not particularly amenable to targeting by small molecules. Another potential target is the ATP binding pocket of JH2. It is conceivable that small molecules that bind here could stabilize the JH2–JH1 autoinhibitory interaction, even in a JH2 mutant (V617F), although such molecules could also have the opposite effect of disrupting the JH2–JH1 autoinhibitory interaction and partially activating JAK2. To what extent a small molecule could reshape the ATP binding pocket in JH2 is also a question, given that the apo and ATP-bound JH2 structures are very similar (26). However, recent studies of compounds that bind in the ATP binding pocket of TYK2 JH2 and inhibit TYK2 signaling provide reason for optimism (45, 46). One senses that a wealth of structural data for JAKs will come to light in the next few years, with implications for novel therapeutic modalities.

## Author Contributions

The author confirms being the sole contributor of this work and approved it for publication.

## Conflict of Interest Statement

The author declares that the research was conducted in the absence of any commercial or financial relationships that could be construed as a potential conflict of interest.
